# Supplementary material for: High-fidelity Cas9-mediated targeting of KRAS driver mutations restrains lung cancer in preclinical models
Source: Nat Commun. 2025 Sep 1;16:7080. doi: 10.1038/s41467-025-62350-4 (PMC12402321; doi:10.1038/s41467-025-62350-4)

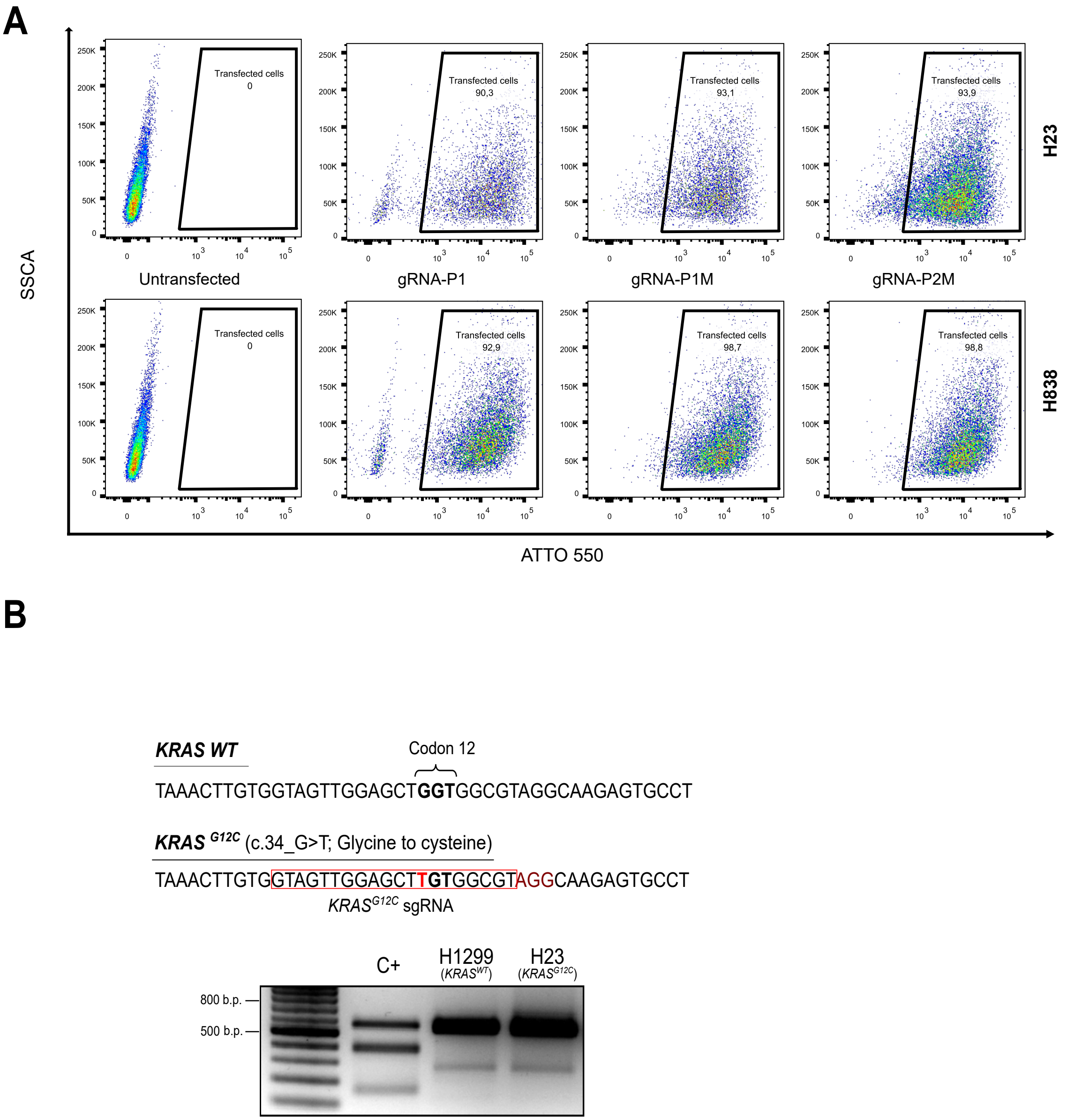

Supplementary figure 1.

(A) Transfection efficiency of the different *KRAS*^{G12C}-mutant-specific RNPs.

Flow cytometry data from the transfections involving *KRAS*^{G12C} sgRNA-containing RNPs, as shown in figure 1C. The Y-axis displays the side scatter values (indicating internal complexity), while the X-axis shows the ATTO 550 signal (Ex 553/Em 575), which is proportional to the internalized RNPs.

(B) Efficiency and specificity of non-HiFi-Cas9 with *KRAS*^{G12C}-specific sgRNA.

T7-endonuclease assay in *KRAS*^{WT} (H1299) and *KRAS*^{G12C} (H23) cell lines.

WT: wildtype, C+: positive control (heteroduplex with indels).

Source data for panel B are provided in the Source Data file.

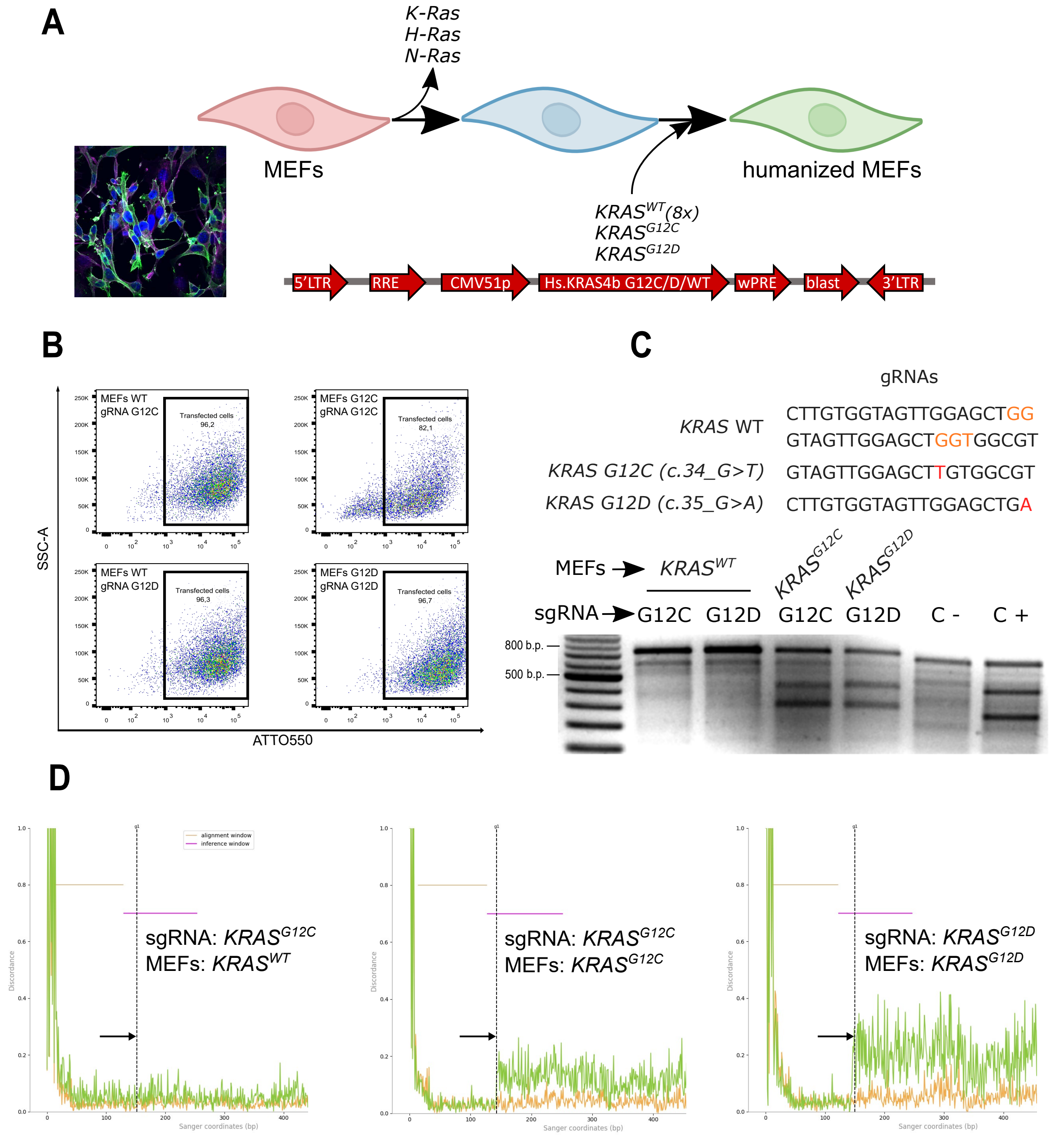

Supplementary figure 2. *KRAS*^{mut} sgRNAs specificity validation in an isogenic model.
(A) Cartoon depicting the generation of the MEF isogenic model.
(B) Dot plots of *KRAS*^{WT}, *KRAS*^{G12C} or *KRAS*^{G12D} MEFs treated with *KRAS*^{mut}-specific sgRNAs.
The X-axis displays the side scatter values (indicating internal complexity), while the Y-axis shows the ATTO 550 signal (Excitation 553/Emission 575), which is proportional to the internalized RNPs.
(C) T7-endonuclease assay on *KRAS*^{WT}, *KRAS*^{G12C} or *KRAS*^{G12D} MEFs treated with *KRAS*$^{G12C/D}$-specific sgRNAs.
C-: negative control. C+: positive control.
(D) Inference of CRISPR Edits (ICE) analysis. The dotted vertical line denotes the DNA cut site (arrow), after which the sequence discrepancy (Y-axis) begins if editing occurs. X-axis displays amplicon coordinates (b.p.).
Source data for panel C are provided in the Source Data file.

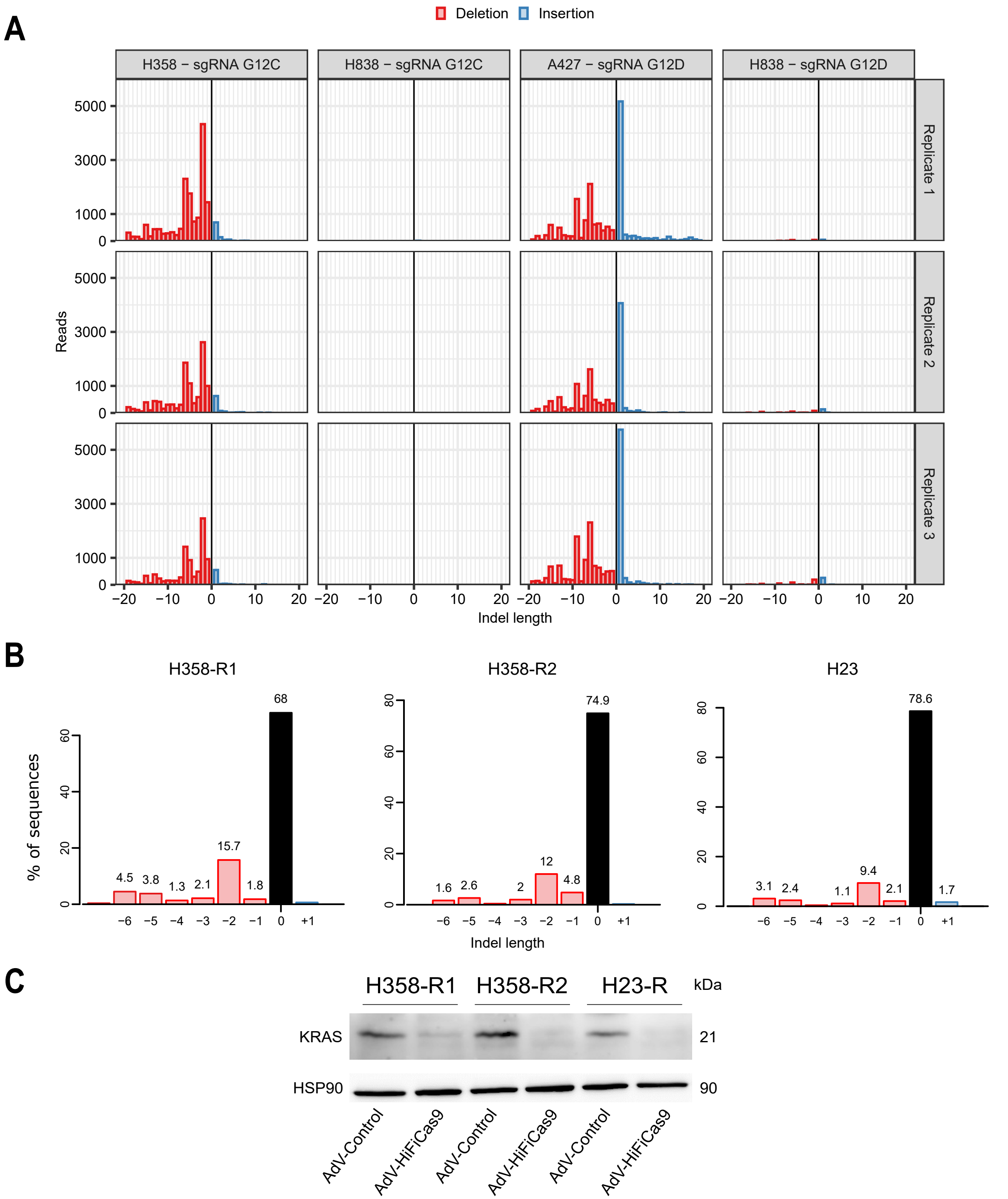

Supplementary figure 3. Edited *KRAS* indel length distribution and expression.

(A) Histogram of the insertions (blue) and deletions (red) in each cell line - sgRNA combination. Y-axis shows the number of reads of the different replicates. X-axis shows indel lenght. Cells were transfected with RNPs contaning sgRNA targeting *KRAS*^{G12C} or *KRAS*^{G12D}.

(B) Indel characterization in sotorasib-resistant cells 72h after AdV-HiFiCas9 transduction. X-axis shows indel lenght.

(C) Western Blot displaying KRAS levels in sotorasib-resistant cells 72h after AdV transduction. Source data for panels C are provided in the Source Data file.

A

Chr	Strand	Start	End	Genomic Hit	Nº of mismatches	Pre-mRNA (Unspliced)	mRNA (CDS)
12	-	25245 341	25245 363	GTAGTTGGAG CT**g**GTGGCGT -AGG	1	*KRAS* ENSG00000133703 **ENST00000311936** **ENST00000256078**	*KRAS* **ENST00000311936** **ENST00000256078**

B

Chr	Strand	Start	End	Genomic Hit	Nº of mismatches	Pre-mRNA (Unspliced)	mRNA (CDS)
19	+	38081 074	38081 096	**tTa**GTGGT**t**GTTG GAGCTGA-TGG	3	*SIPA1L3* (ENSG00000105738) **(ENST00000222345)**	No data
3	+	10083 2335	10083 2357	**g**TTGT**t**GT**t**GTTG GAGCTGA-AGG	3	*ABI3BP* (ENSG00000154175) **(ENST00000284322)** **(ENST00000495063)**	*ABI3BP* ENST00000495063
12	-	25245 341	25245 363	CTTGTGGTAGTT GGAGCTG**g**-TGG	1	*KRAS* ENSG00000133703 **ENST00000311936** **ENST00000256078**	*KRAS* **ENST00000311936** **ENST00000256078**

C

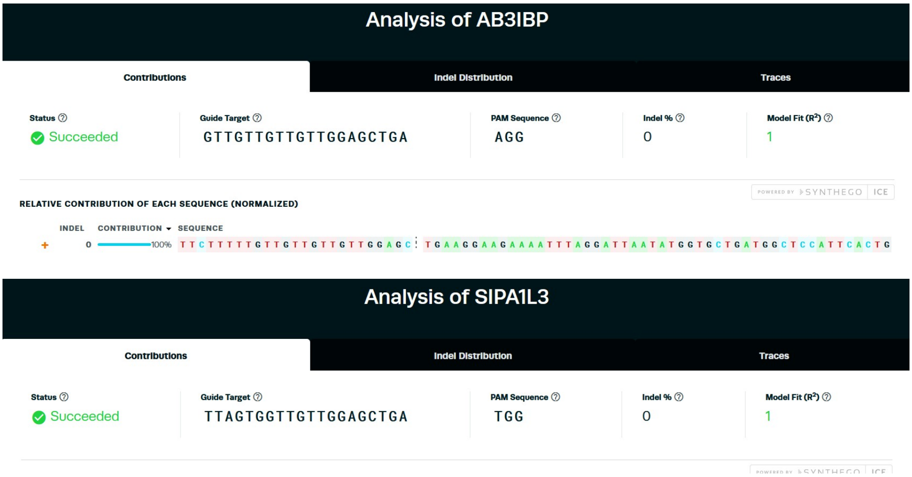

Supplementary figure 4. Off-target prediction for sgRNAs targeting *KRAS*^{G12C} and *KRAS*^{G12D} in H838 and H358 cells.
(A) Off-target prediction of Off-Spotter and Cas-OFFinder algorithms for sgRNA-*KRAS*^{G12C}.
(B) Off-target prediction of Off-Spotter and Cas-OFFinder algorithms for sgRNA-*KRAS*^{G12D}.
(C) Sanger sequencing results of candidate genes: *AB3IBP* (above) and *SIPA1L3* (below) analyzed with ICE software.

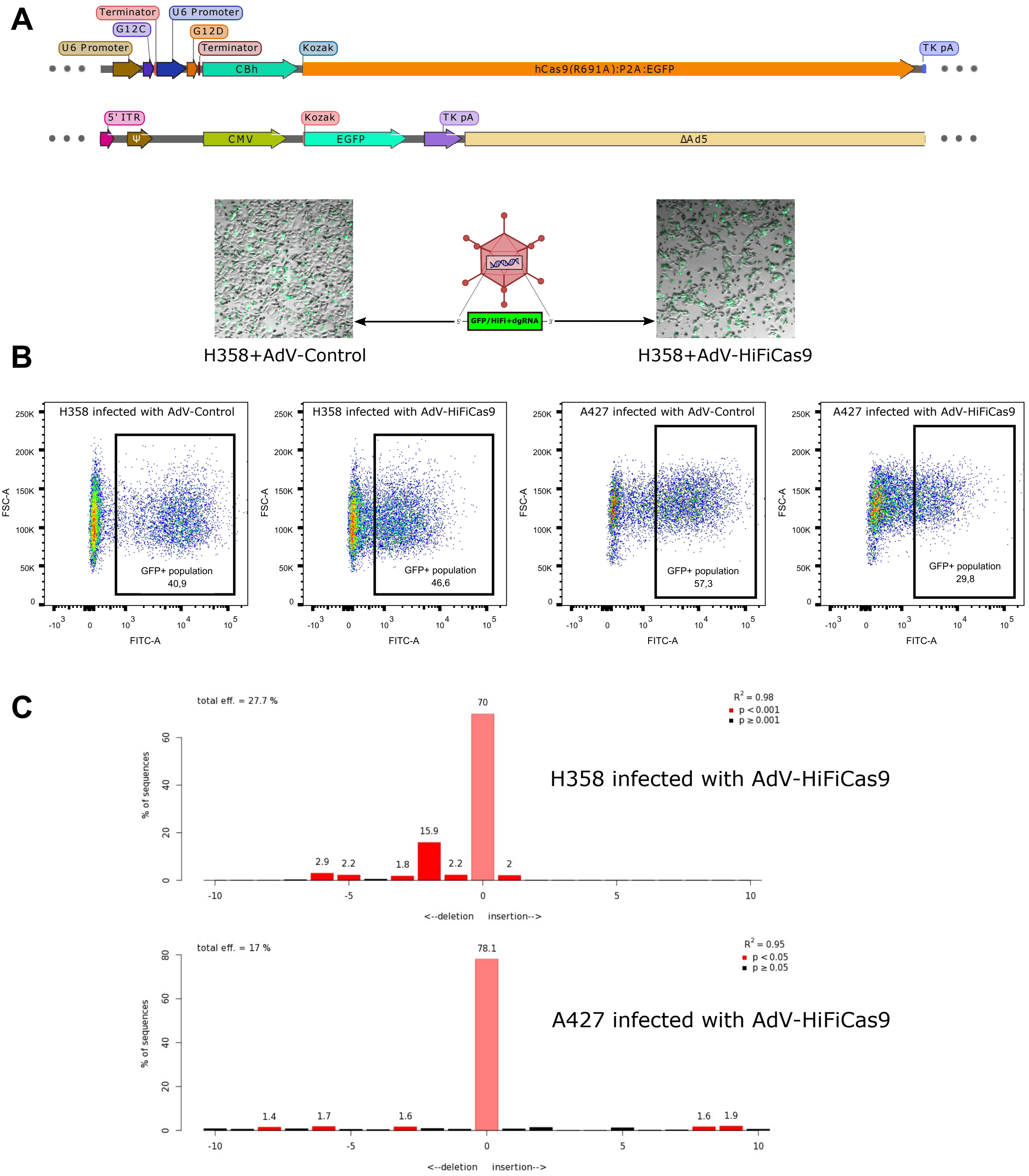

Supplementary figure 5. Adenovirus design validation.

(A) Design of the doble guide RNA (*KRAS*^{G12C} and *KRAS*^{G12D})-HiFi-Cas9-GFP and control GFP vectors.

(B) Infection efficiency of both adenoviral particles was assessed. The X-axis displays forward scatter values (indicating cell size), while the Y-axis shows the GFP fluorescence signal (Ex 488 nm/Em 509 nm), which is proportional to the number of transduced cells.

(C) Editing and indel distribution in H358 and A427 cell lines. X-axis shows indel length.

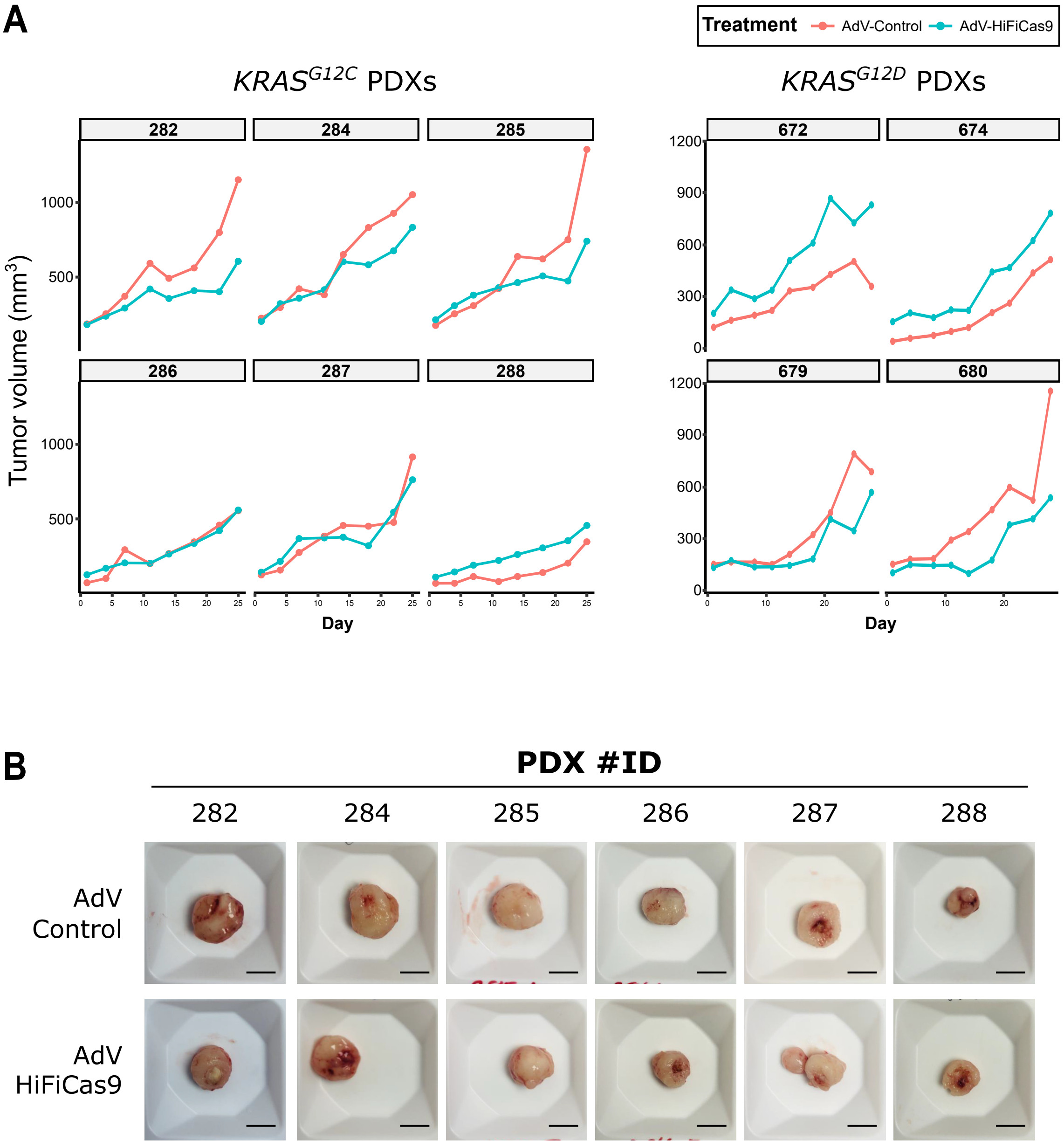

Supplementary figure 6. Impact of mutant *KRAS* editing on tumor growth inhibition in PDX models.
(A) Tracking individual tumor volumes (mm^3) over time in treated $KRAS^{mut}$ PDX models.
Left panel: $KRAS^{G12C}$ PDX model. Right panel: $KRAS^{G12D}$ PDX model.
(B) *Ex vivo* images of $KRAS^{G12C}$ tumors extracted 28 days post-treatment. Scale bar=10 mm.
Upper row: Control-treated $KRAS^{G12C}$ PDX tumors.
Bottom row: HiFiCas9-treated $KRAS^{G12C}$ PDX tumors.

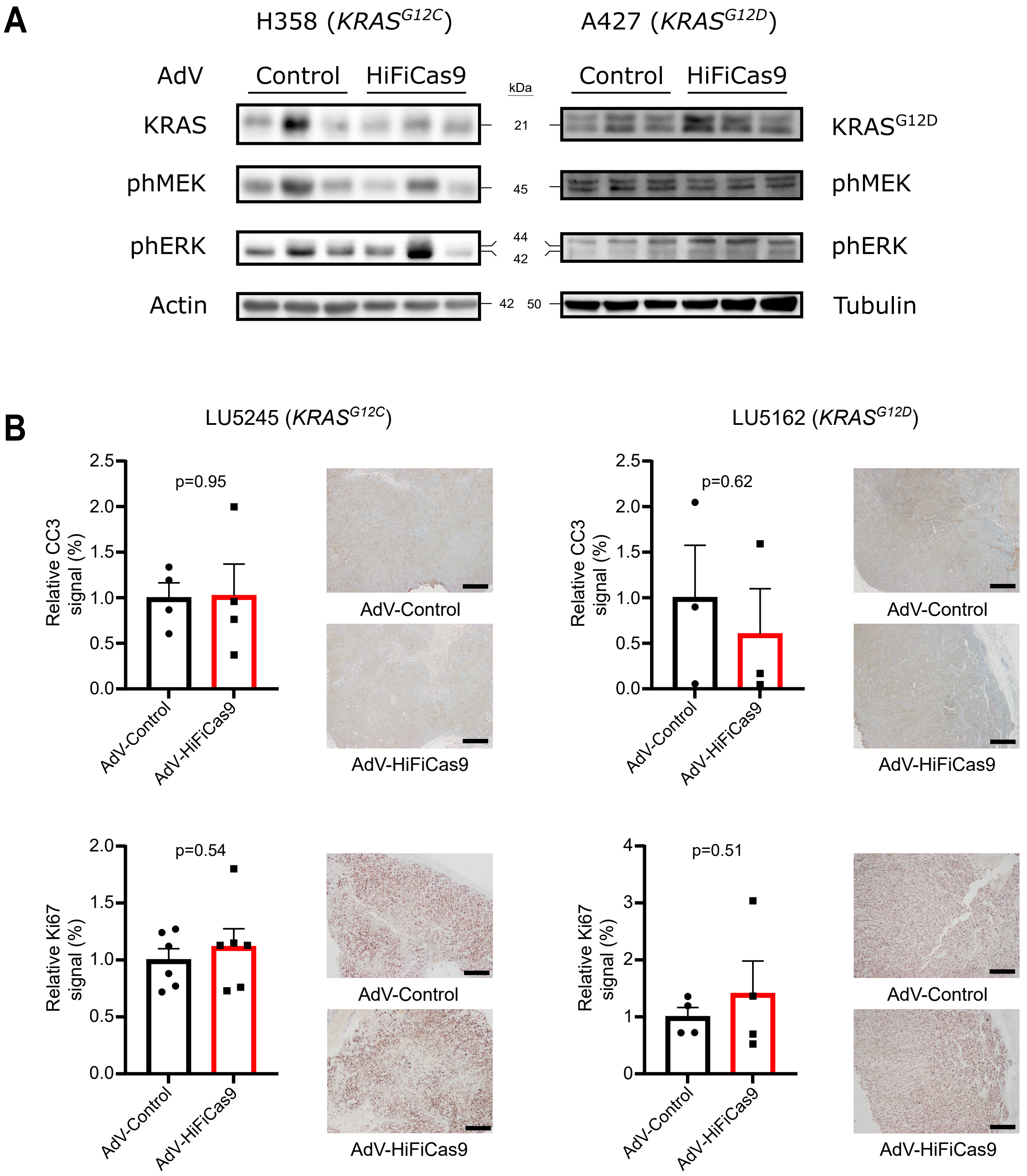

Supplementary figure 7. Xenografs characterization.

(A) *Ex vivo* immunoblots of *KRAS*$^{G12C/D}$ CDX tumors extracted 60 days post-treatment.

(B) Immunohistochemical analysis of *KRAS*^{G12C} (left) and *KRAS*^{G12D} (right) PDX models.

Values represent the mean ± SEM of N independent PDX samples, normalized to the AdV-Control group. (LU5162: Ki67, N=4; CC3, N=3. LU5245: Ki67, N=6; CC3, N=4). Statistical analysis was performed using an unpaired two-tailed t-test.

Upper: Cleaved Caspase 3 (CC3). Bottom: Ki67. Scale bar = 500 µm.

Source data for panels A and B are provided in the Source Data file.

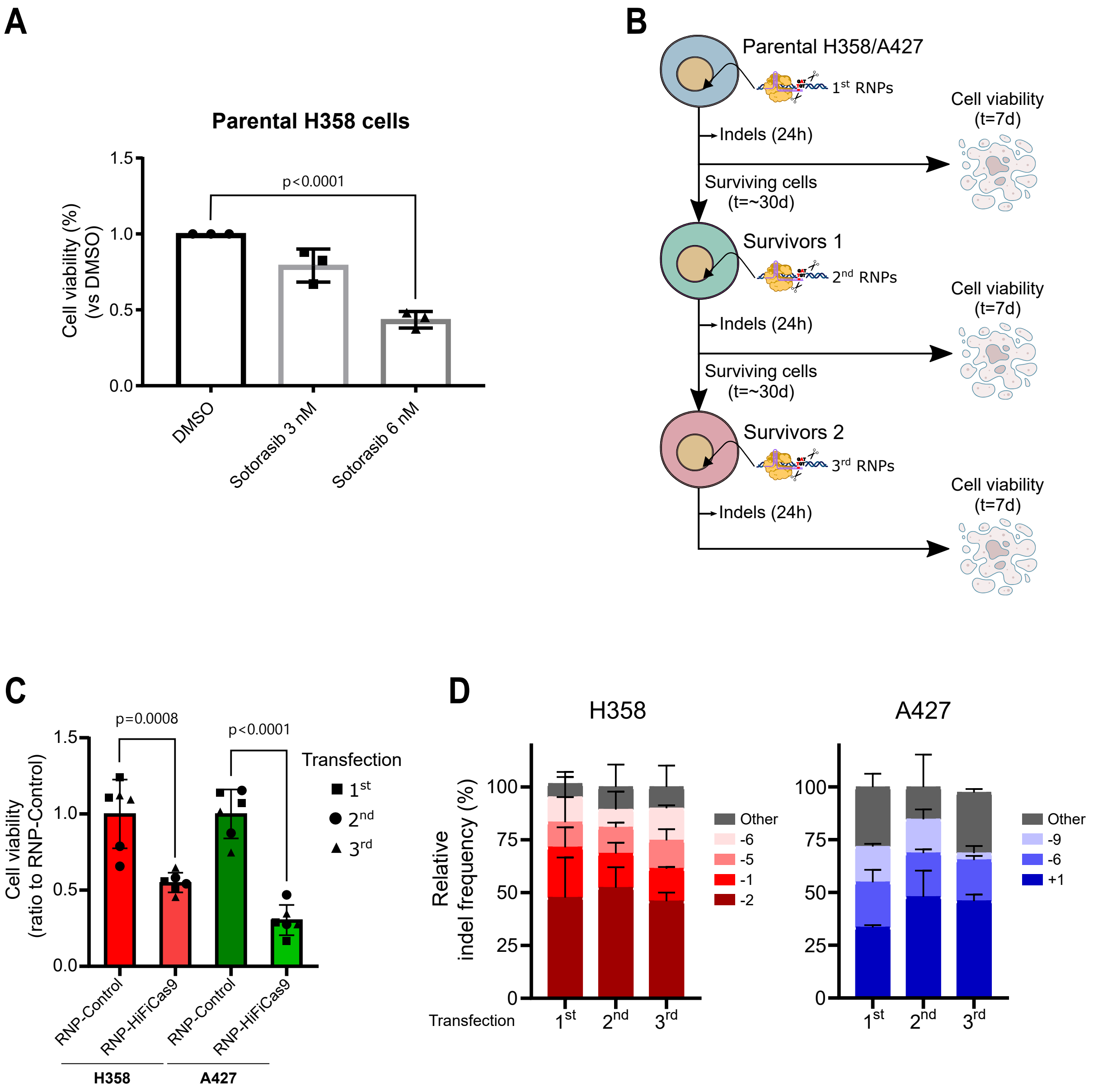

Supplementary figure 8. Sotorasib treatment in parental cells and analysis of potential resistance mechanisms.

(A) Cell viability assay to test sotorasib activity in parental H358 cells. Data represent mean ± SEM from three independent biological replicates, analyzed using an unpaired two-tailed t-test.

(B) Graphical representation of the experimental design to capture potential resistance or adpatation mechanisms.

(C) Cell viability at consecutive rounds of RNPs transfection in H358 and A427 parental and surviving cells. Data represent mean ± SD from six independent biological replicates, analyzed using an unpaired two-tailed t-test.

(D) Indel distribution in each of the three rounds of transfection in H358 and A427 parental and surviving cells. Data represent mean ± SEM from two independent biological replicates.

Source data for panels A, C and D are provided in the Source Data file.

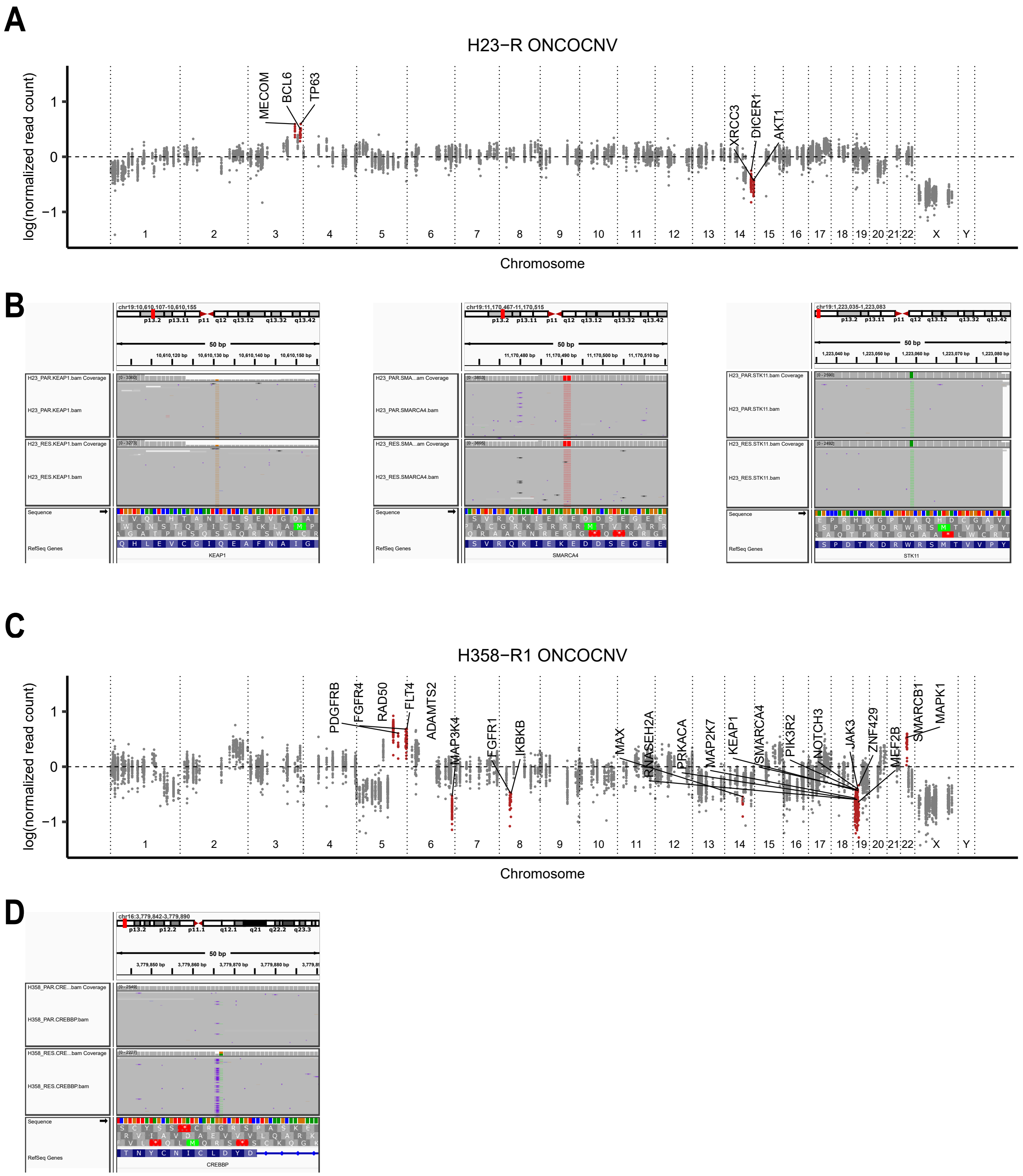

Supplementary figure 9. Genetic characterization of Sotorasib-resistant cell lines by targeted sequencing.

(A, C) Copy number variation (CNV) analysis of (A) H23-R and (C) H358-R1 cell lines.

Significant CNV calls (q < 0.01, CN > 2.5 or < 1.5) identified by ONCOCNV are highlighted in red.

Each dot represents an amplicon from the sequencing panel.

(B, D) Integrated Genomics Viewer (IGV) screenshots of BAM files.

(B) KEAP1 (p.Q193H), SMARCA4 (p.K1566_E1567delinsNTer) and STK11 (p.W332Ter) variants in both parental and H23-R cells.

(D) CREBBP frameshift variant (p.L1728fs) called in H358-R1 by Mutect2 and PipeIT2.

Supplementary Figure 1B

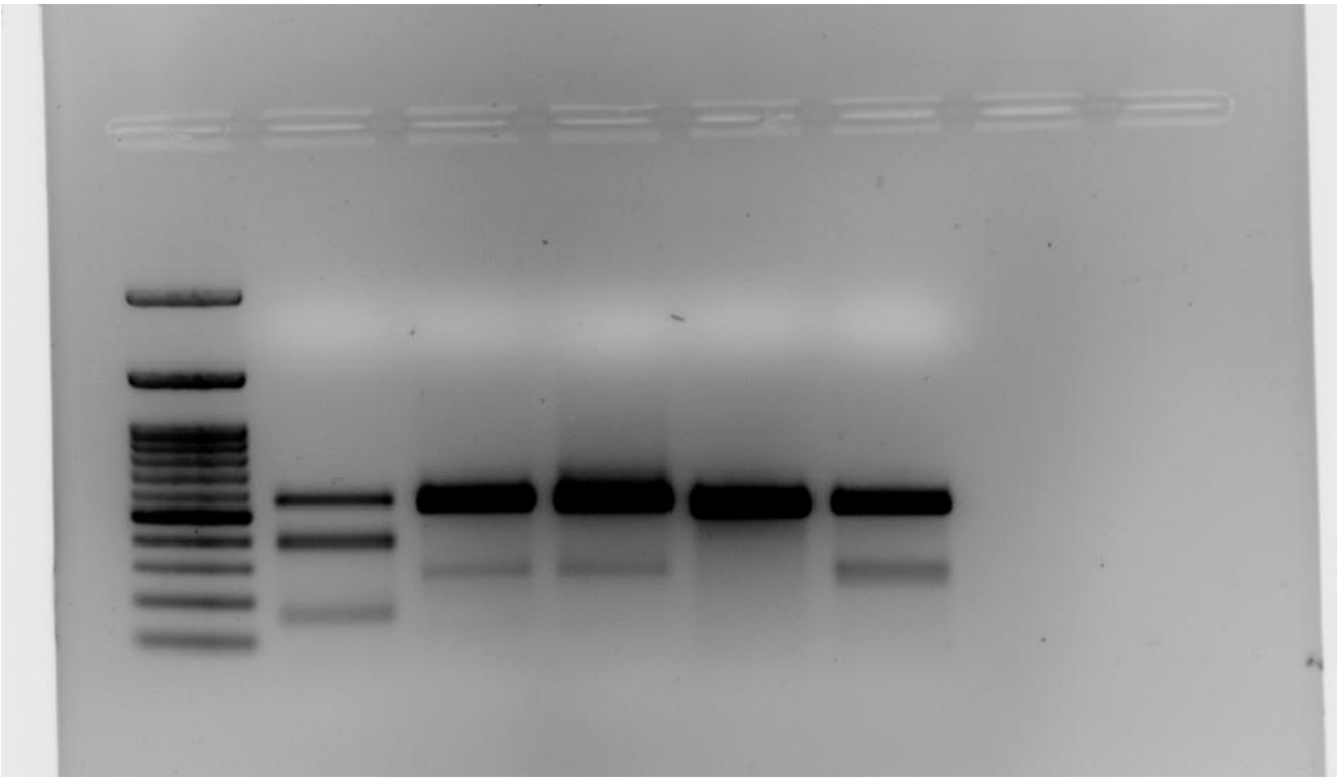

Supplementary Figure 2C

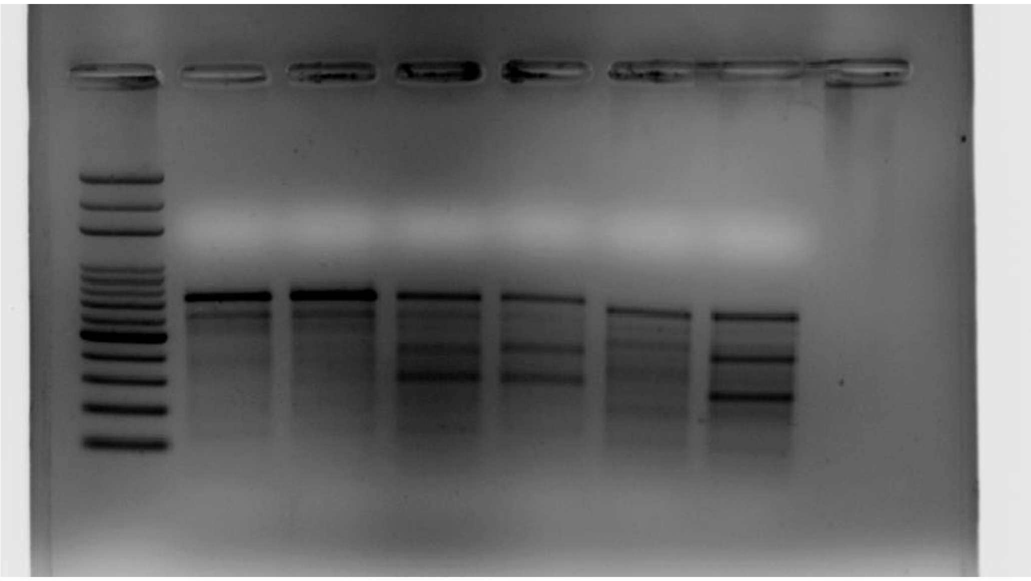

Supplementary Figure 3C

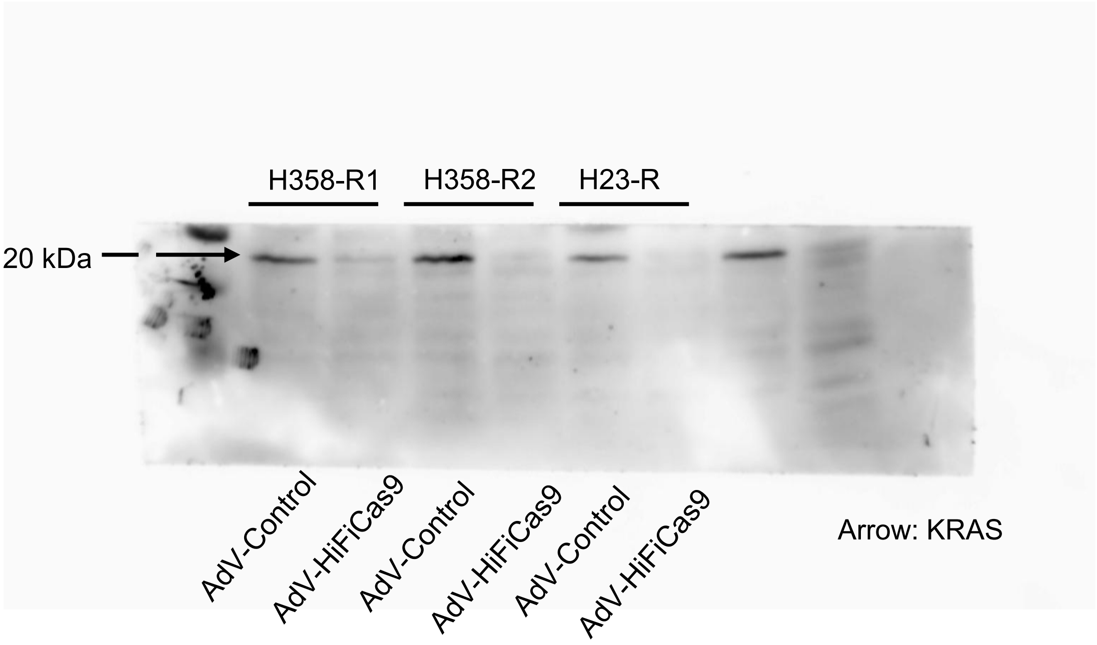

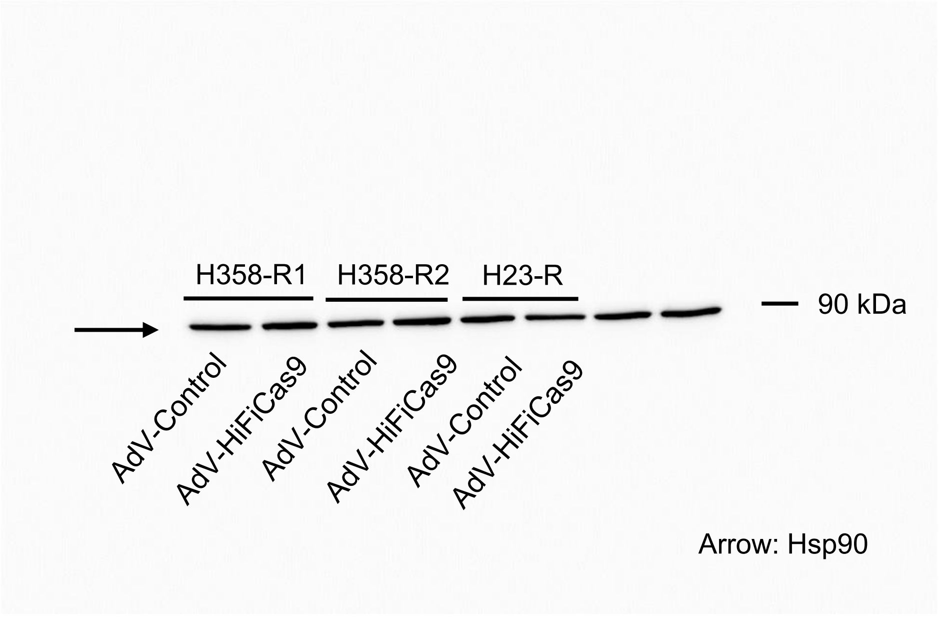

Supplementary Figure 7A

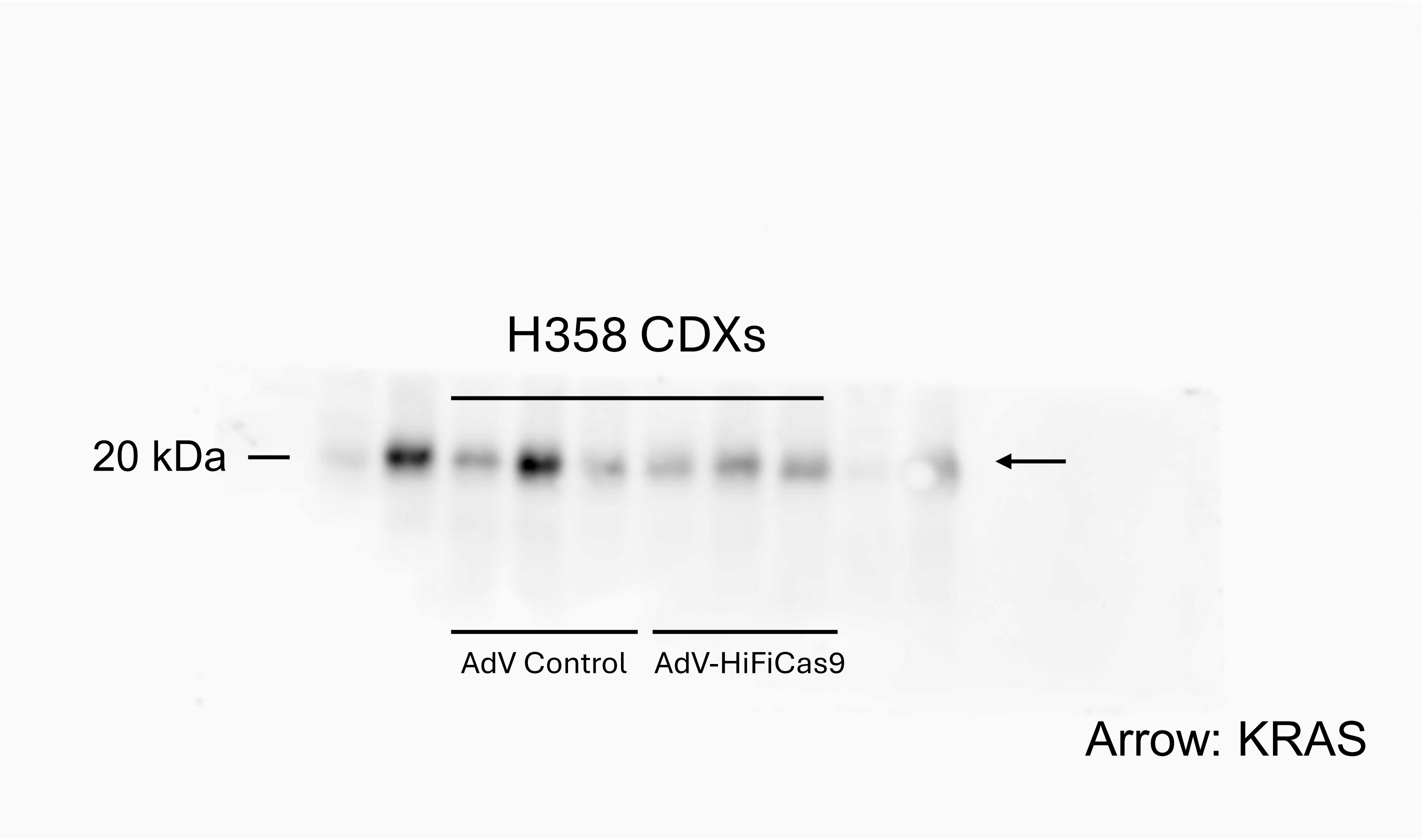

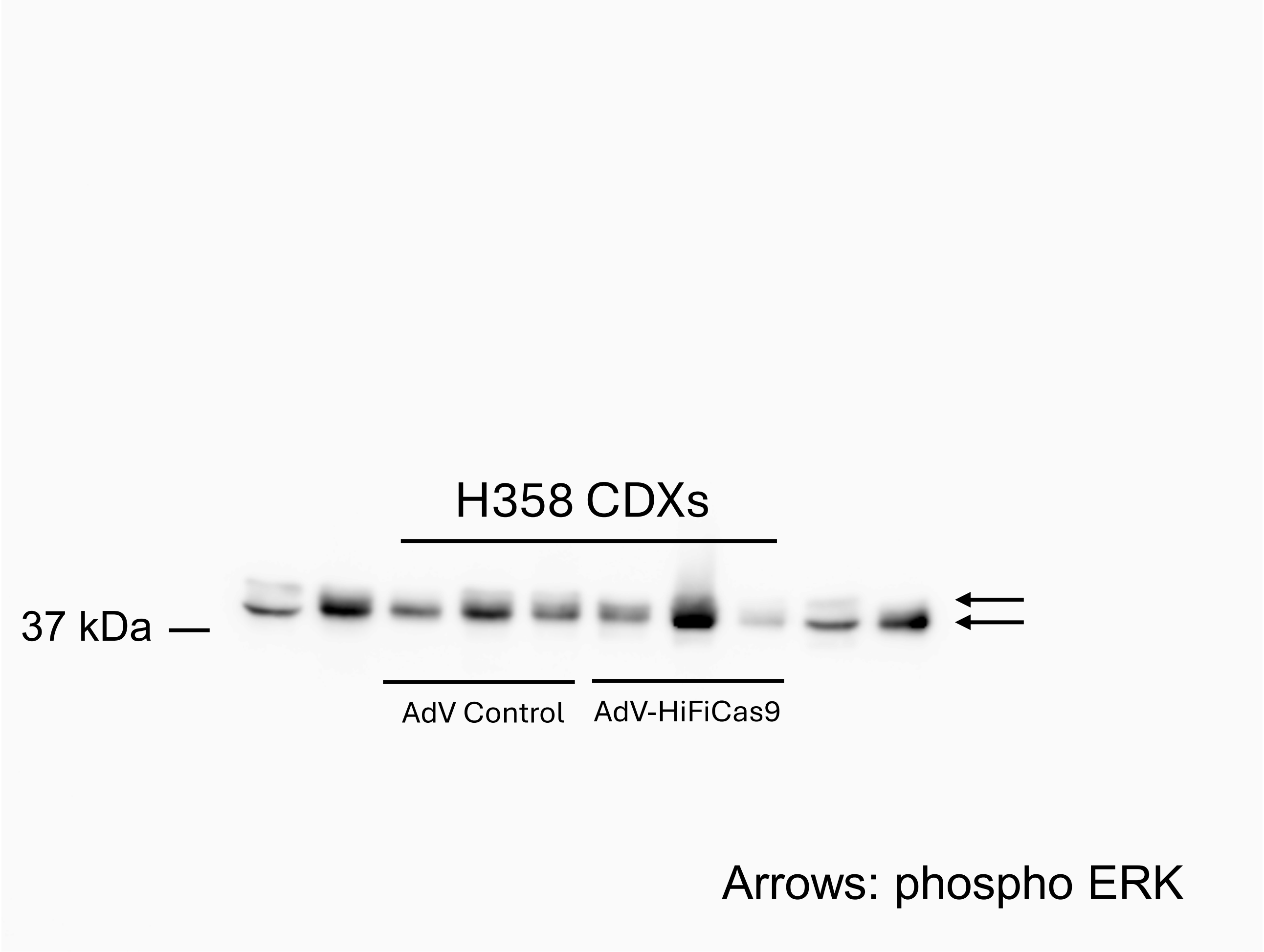

Supplementary Figure 7A

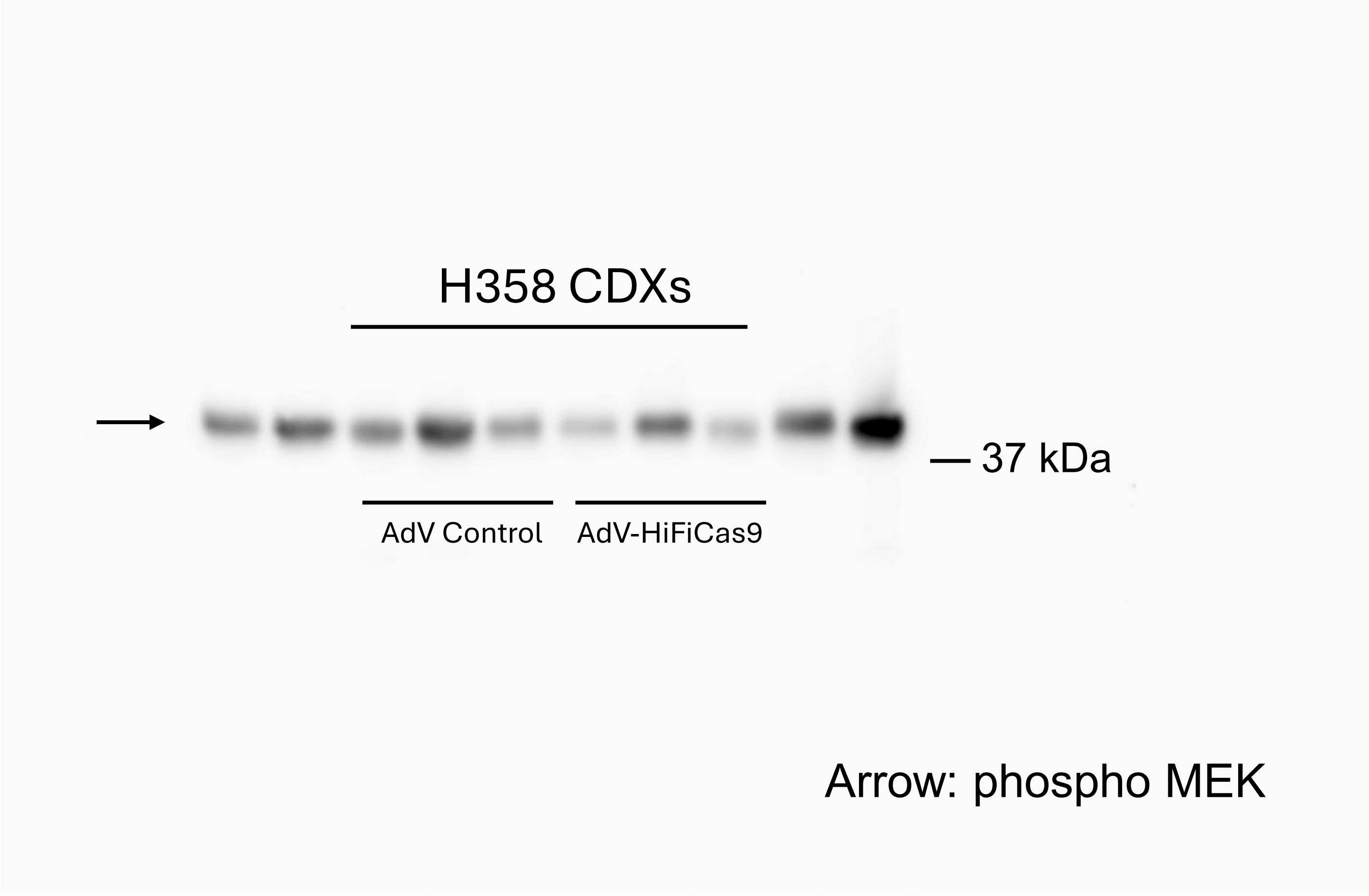

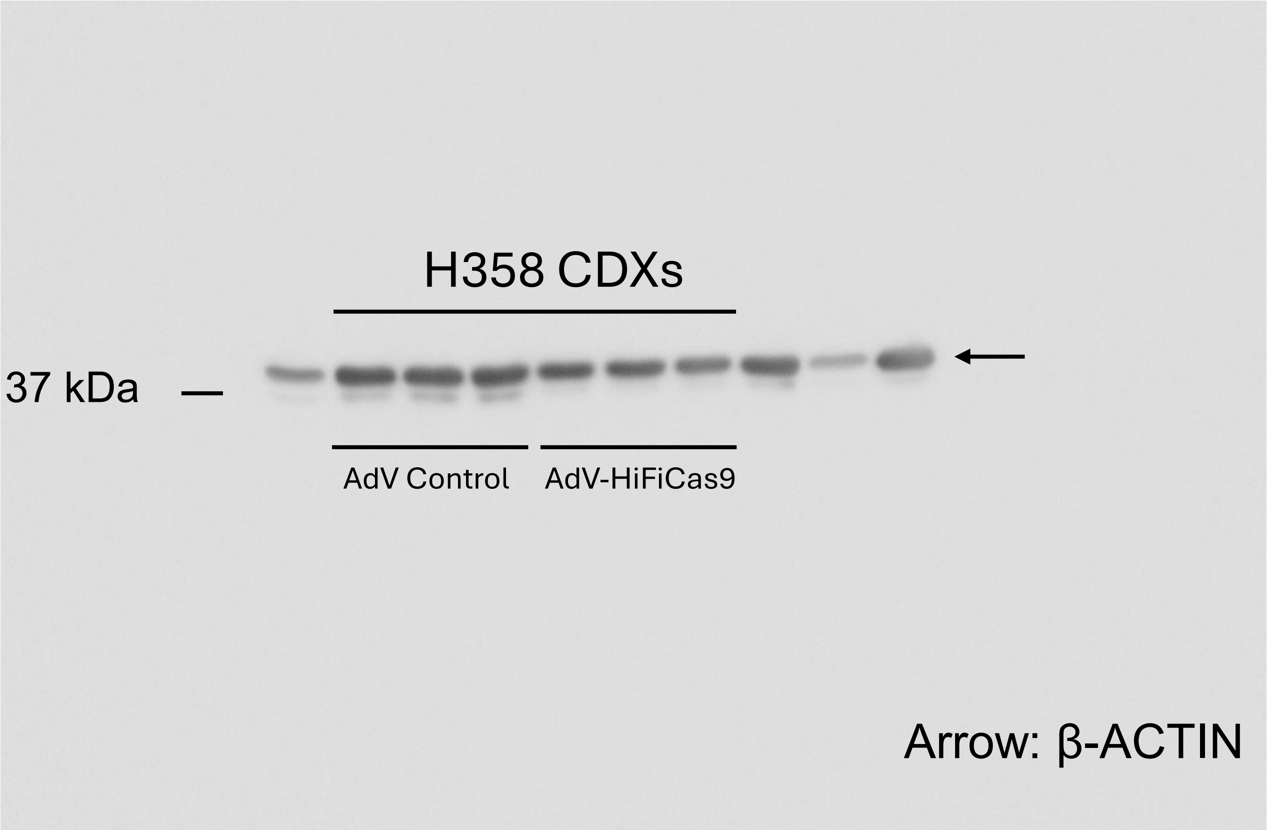

Supplementary Figure 7A

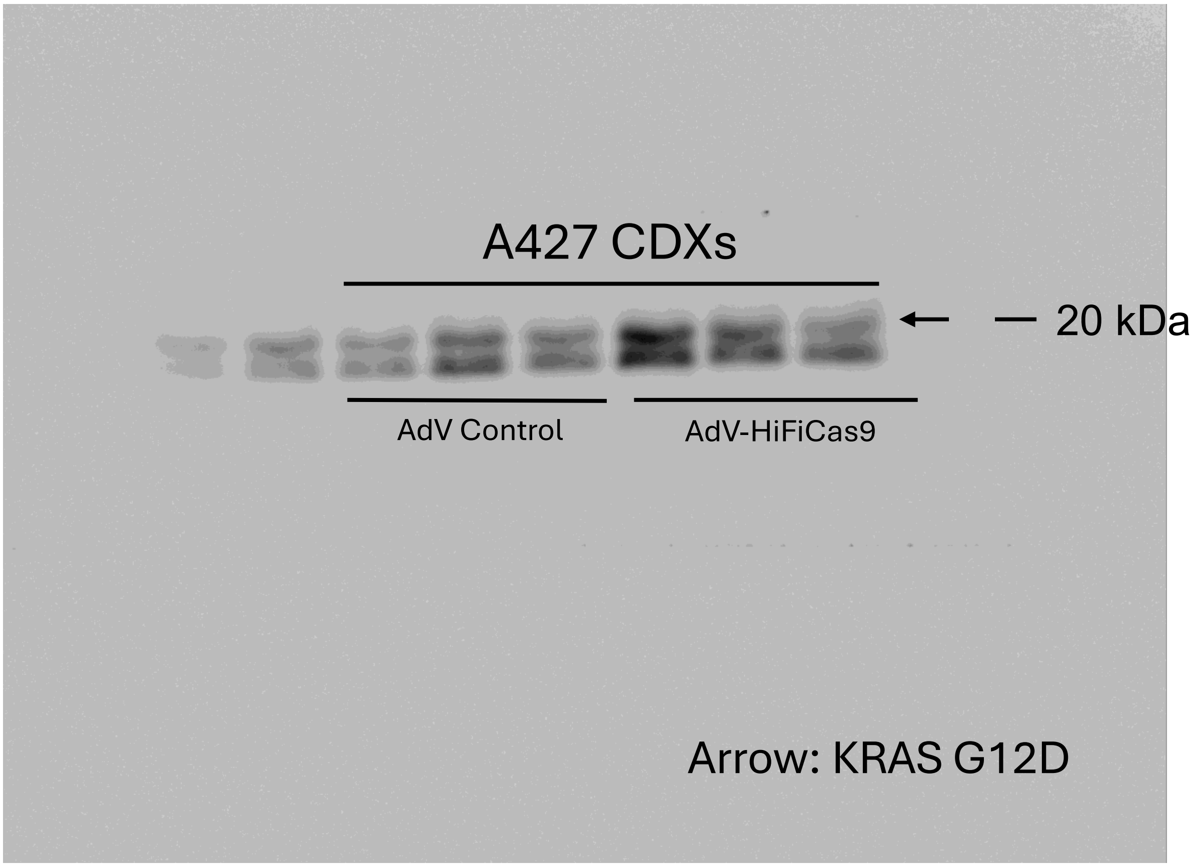

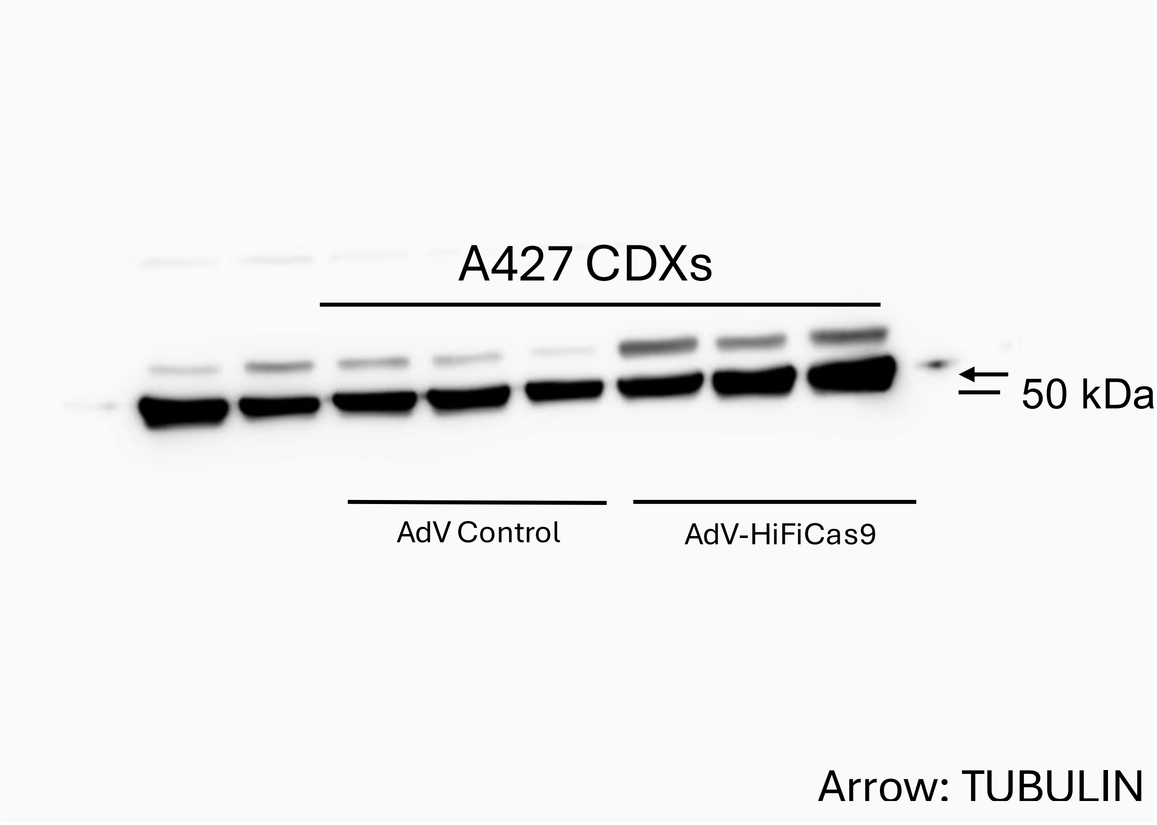

Supplementary Figure 7A

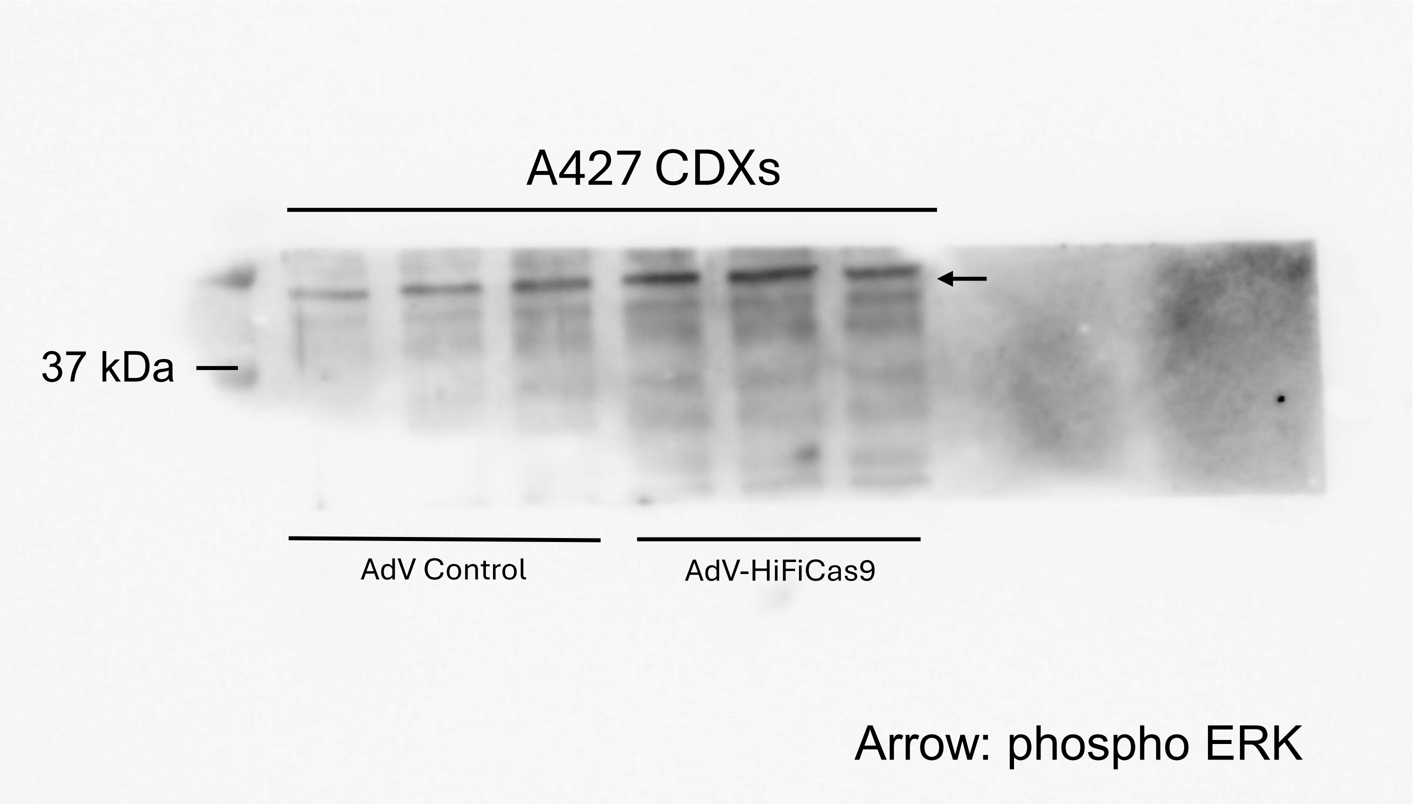

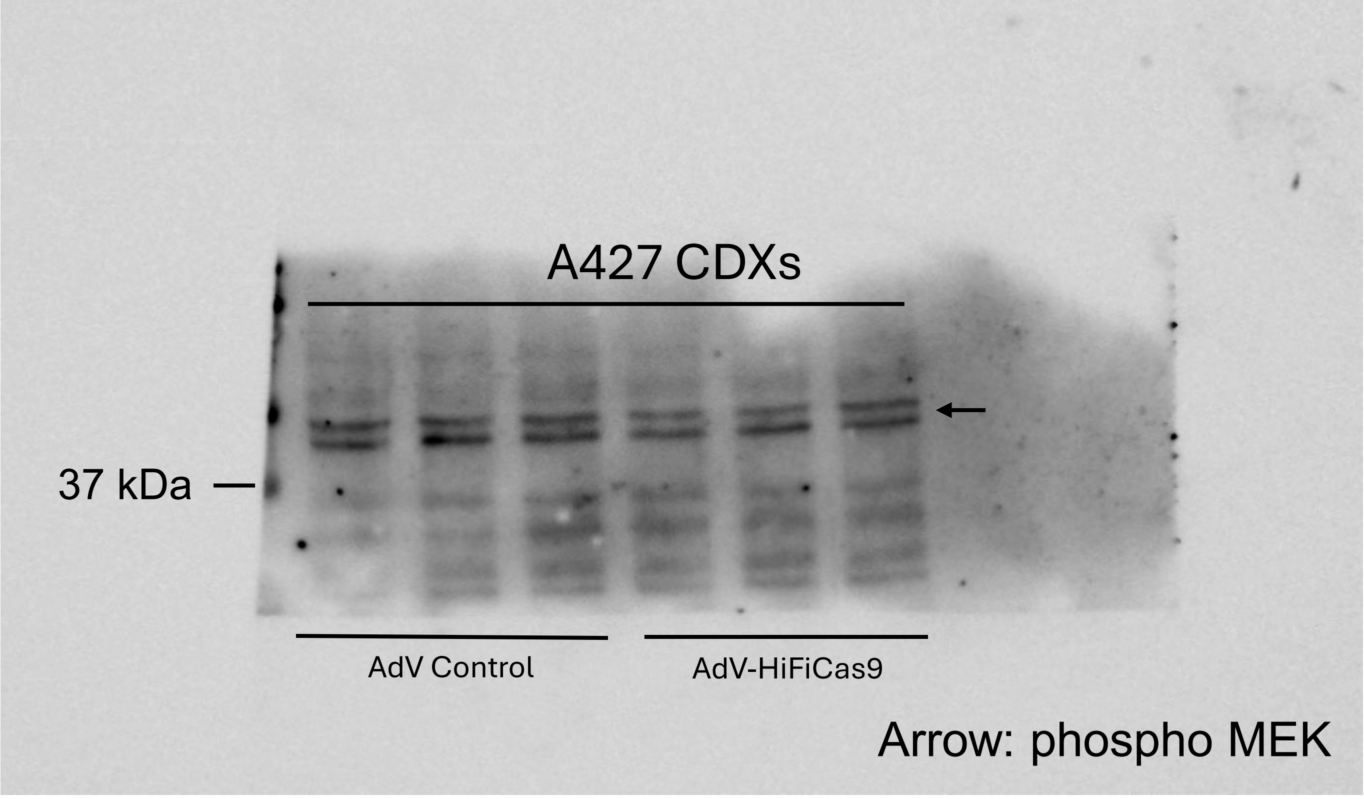

Supplementary Figure 7A

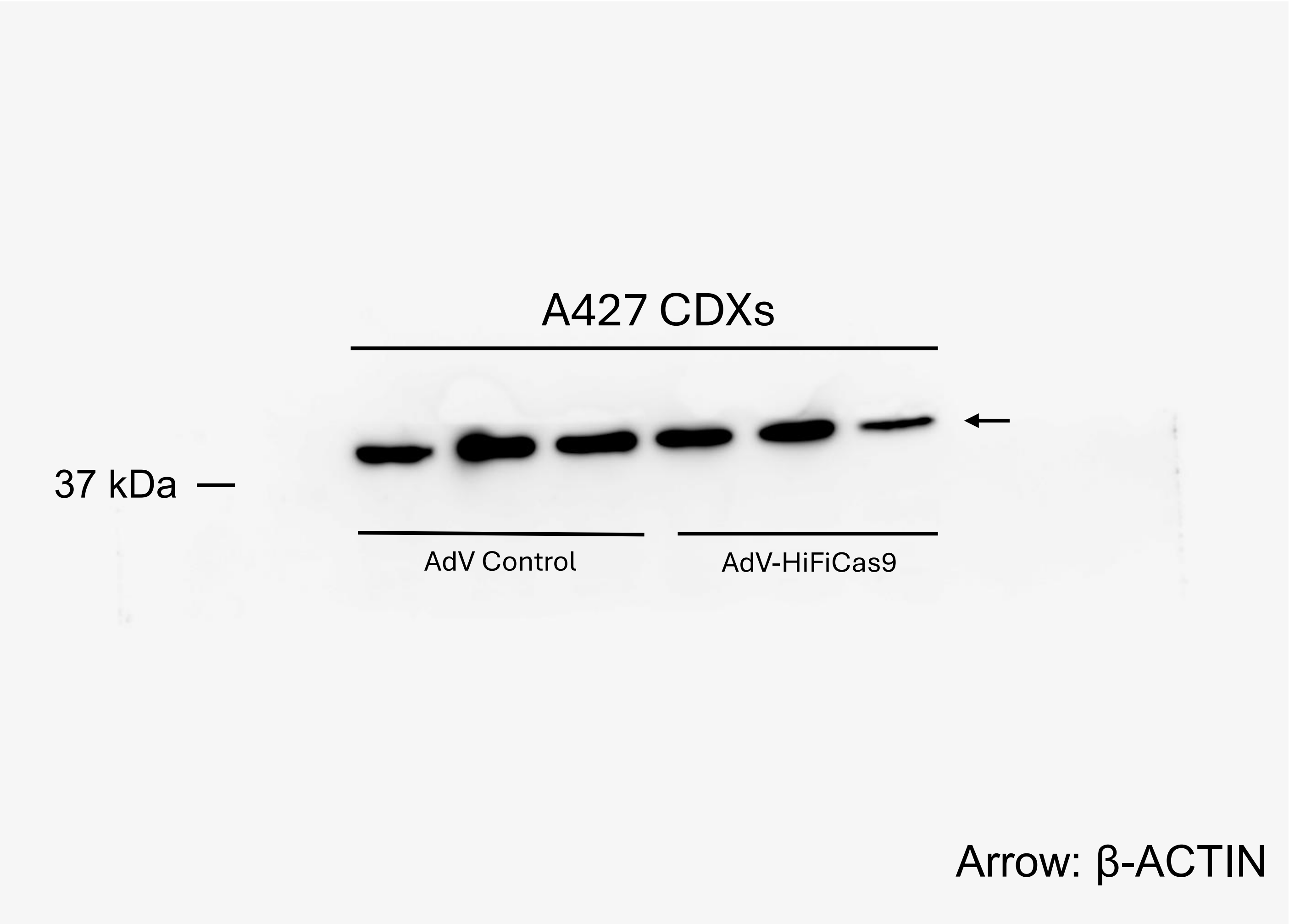

Supplement: Supplementary file 1 — Supplementary Information [file 41467_2025_62350_MOESM1_ESM.pdf]
