# Supplementary material for: High-fidelity Cas9-mediated targeting of KRAS driver mutations restrains lung cancer in preclinical models
Source: Nat Commun. 2025 Sep 1;16:7080. doi: 10.1038/s41467-025-62350-4 (PMC12402321; doi:10.1038/s41467-025-62350-4)
Supplement: Supplementary file 3 — Reporting Summary [file 41467_2025_62350_MOESM3_ESM.pdf]

Corresponding author(s): Pedro Medina Vico

Last updated by author(s): Jul 9, 2025

Reporting Summary

Nature Portfolio wishes to improve the reproducibility of the work that we publish. This form provides structure for consistency and transparency in reporting. For further information on Nature Portfolio policies, see our Editorial Policies and the Editorial Policy Checklist.

Statistics

For all statistical analyses, confirm that the following items are present in the figure legend, table legend, main text, or Methods section.

n/a	Confirmed	
☐	☒	The exact sample size (n) for each experimental group/condition, given as a discrete number and unit of measurement
☐	☒	A statement on whether measurements were taken from distinct samples or whether the same sample was measured repeatedly
☐	☒	The statistical test(s) used AND whether they are one- or two-sided<br>*Only common tests should be described solely by name; describe more complex techniques in the Methods section.*
☒	☐	A description of all covariates tested
☐	☒	A description of any assumptions or corrections, such as tests of normality and adjustment for multiple comparisons
☐	☒	A full description of the statistical parameters including central tendency (e.g. means) or other basic estimates (e.g. regression coefficient) AND variation (e.g. standard deviation) or associated estimates of uncertainty (e.g. confidence intervals)
☐	☒	For null hypothesis testing, the test statistic (e.g. F, t, r) with confidence intervals, effect sizes, degrees of freedom and P value noted<br>*Give P values as exact values whenever suitable.*
☐	☒	For Bayesian analysis, information on the choice of priors and Markov chain Monte Carlo settings
☒	☐	For hierarchical and complex designs, identification of the appropriate level for tests and full reporting of outcomes
☒	☐	Estimates of effect sizes (e.g. Cohen's d, Pearson's r), indicating how they were calculated

Our web collection on statistics for biologists contains articles on many of the points above.

Software and code

Policy information about availability of computer code

Data collection	For Western blot imaging: ImageQuant LAS-4000, using chemoluminiscence automatic exposure time.<br>For flow cytometry: BD FacsCanto II.<br>For Cell viability assays, CellTiter-Glo luminescent-based assay was measured in the Promega GloMax® Explorer Multimode Microplate Reader.<br>For agarose gel imaging: AVEGENE Slite-140R Transilluminator.<br>Immunostainings were performed using standard procedures by AtrysHealth SA (Barcelona, Spain). Representative images were taken using Olympus BX43 microscope (Olympus Life Science, MA, USA).<br>For Next-generation sequencing (NGS) analysis, sequencing was performed on a paired-end Illumina platform, generating 250 bp paired-end raw reads. Quantified libraries were pooled and sequenced on the Illumina NovaSeq6000 platform, aiming for a minimum of 30,000 paired-end reads per sample.<br>Filtering and analysis methods:<br>a. Filtering out the adapter sequence and barcode and primer sequence in reads.<br>b. Use Flash(Mago, T., et al, 2011) software to combine the read pairs with overlap as Tags.<br>c. Fastp(Bokulich NAetal.,2012) software is used to filter the combined data, and the sequences containing more N or more low-quality bases are filtered out.<br>d. Filter out the chimeric sequences in the combined sequence(Edgar RC etal.,2011).
Data analysis	Relative intensity of Western blot, agarose gel bands and immunostains were measured/quantified using ImageJ software.<br>Flow cytometry data were processed using FlowJo software v10.7.<br>Graphs and statistical analysis were performed using Microsoft Excel 2022, GraphPad Prism v9.0 and R version 4.0.2.<br>For NGS data, paired-end reads were demultiplexed by sample barcodes, followed by trimming of barcode and primer sequences.

Overlapping paired-end reads were merged using FLASH and further processed for quality filtering with fastp software.
Quantification of KRAS editing from preprocessed FASTQ files was performed with CRISPResso2 in single-end mode with default amplicon end trimming disabled. Allele edition frequencies were recalculated in R version 4.0.2, including only those edits supported by at least 10 reads. Reads tagged as "ambiguous" by CRISPResso2 were classified as edited mutant alleles, as the loss of codon 12 is primarily attributable to editing events specific to mutant alleles, with negligible occurrences in KRAS WT cell lines.
Inkscape was used for figure arrangement and image construction throughout the manuscript

For manuscripts utilizing custom algorithms or software that are central to the research but not yet described in published literature, software must be made available to editors and reviewers. We strongly encourage code deposition in a community repository (e.g. GitHub). See the Nature Portfolio guidelines for submitting code & software for further information.

Data

Policy information about availability of data

All manuscripts must include a data availability statement. This statement should provide the following information, where applicable:

- Accession codes, unique identifiers, or web links for publicly available datasets
- A description of any restrictions on data availability
- For clinical datasets or third party data, please ensure that the statement adheres to our policy

Targeted-sequencing raw data generated in this study have been deposited into the SRA database under accession number PRJNA965929 [https://www.ncbi.nlm.nih.gov/bioproject/PRJNA965929/] (Characterization of indels in Cas9-induced KRAS edition in NSCLC cells) without access restrictions

Research involving human participants, their data, or biological material

Policy information about studies with human participants or human data. See also policy information about sex, gender (identity/presentation), and sexual orientation and race, ethnicity and racism.

Reporting on sex and gender	Selection of human PDXs, PDXOs, or derived cell lines for this study was based on KRAS mutational status and not on the sex/gender or race/ethnicity of the patient of origin, as there is no evidence of sex-related differences in the molecular mechanisms driven by oncogenic KRAS.
Reporting on race, ethnicity, or other socially relevant groupings	Selection of human PDXs, PDXOs or derived cell lines for this study was based on KRAS mutational status and not the race/ethnicity of the patient of origin, as there is no evidence of race-related differences in the molecular mechanisms driven by oncogenic KRAS.
Population characteristics	No population characteristics were considered
Recruitment	No patients were recruited for this study
Ethics oversight	The CrowBio PDX experiments were performed under the Institutional Animal Care and Use Committee (IACUC)-approved protocol CBSD-ACUP-001. All procedures complied with the U.S. Department of Agriculture's Animal Welfare Act (9 CFR Parts 1, 2, and 3), the Association for Assessment and Accreditation of Laboratory Animal Care (AAALAC) standards, and Crown Bioscience San Diego's internal Standard Operating Procedures.<br>For the PDXO models, PDXs were established at the Seville Institute of Biomedicine (IBIS) and Spanish National Cancer Research Center (CNIO) using lung cancer samples from patients from Virgen del Rocío Hospital (Seville) and 12 de Octubre Hospital (Madrid). The research project was approved by the hospitals' ethics committees (Approval ID:2012PI/241 (Seville) and CEIm 20/090 (Madrid)). All patients provided written informed consent according to the protocol approved by the local ethics committee. All animal studies were performed according to animal care guidelines and approved by the Consejería de Agricultura of the Junta de Andalucía (Approval ref: SSA/SI/MD/pdm) and by Animal Protection of the Comunidad Autónoma de Madrid (Approval ID: PROEX 084/15, PROEX 313/19, PROEX 297.5/22).

Note that full information on the approval of the study protocol must also be provided in the manuscript.

Field-specific reporting

Please select the one below that is the best fit for your research. If you are not sure, read the appropriate sections before making your selection.

☒ Life sciences ☐ Behavioural & social sciences ☐ Ecological, evolutionary & environmental sciences

For a reference copy of the document with all sections, see nature.com/documents/nr-reporting-summary-flat.pdf

Life sciences study design

All studies must disclose on these points even when the disclosure is negative.

Sample size	Sample sizes are specified in the methods section and the pertinent figure and figure legend. Sample sizes were estimated according to similar experiments performed in previously published studies (at least, n=3 for cell line experiments; and n=4 for mice experiments).
Data exclusions	No data was excluded

Replication	Independent biological replicates were routinely carried out. The number of biological replicates (n) is noted in every experiment and the methods section. All attempts at replication were successful.
Randomization	For all the mice experiments, mice were randomly allocated to each experimental group
Blinding	Treatment administration to PDXs was performed blindly by staff at Crown Bioscience.<br>Treatment administration to PDXOs was performed blindly.

Reporting for specific materials, systems and methods

We require information from authors about some types of materials, experimental systems and methods used in many studies. Here, indicate whether each material, system or method listed is relevant to your study. If you are not sure if a list item applies to your research, read the appropriate section before selecting a response.

Materials & experimental systems

n/a	Involved in the study	
☐	☒	Antibodies
☐	☒	Eukaryotic cell lines
☒	☐	Palaeontology and archaeology
☐	☒	Animals and other organisms
☒	☐	Clinical data
☒	☐	Dual use research of concern
☒	☐	Plants

Methods

n/a	Involved in the study	
☒	☐	ChIP-seq
☐	☒	Flow cytometry
☒	☐	MRI-based neuroimaging

Antibodies

Antibodies used	Anti-KRAS (Santa Cruz, #SC-30, (Dilution 1:200)), anti-KRAS-G12D (Cell Signaling, 168 #14429S (Dilution 1:1.000)), anti-phERK (Cell Signaling, #4370T(Dilution 1:1.000)), anti-phAKT (Cell Signaling, #4060T, (Dilution 1:1.000)), anti-phP70S6 (Santa Cruz, #SC-8416, (Dilution 1:200)), anti-β-actin (Merck, #A5441, (Dilution 1:10.000)), and anti-HSP90 (Cell Signaling, #4877, (Dilution 1:1.000)) antibodies
Validation	All used antibodies are Western grade and validated by manufacturer.

Eukaryotic cell lines

Policy information about cell lines and Sex and Gender in Research

Cell line source(s)	NCI-H1299, NCI-H358, NCI-H23, NCI-H1792, NCI-H2122, A427, NCI-H838, and SKLU-1 were purchased from the American Type Culture Collection (ATCC).<br>Ras initiative team kindly donated us the KRASWT (#RPZ25854), KRASG12C (#RPZ26186), and KRASG12D (#RPZ26198) MEF variants.
Authentication	All cell lines were authenticated by the vendor (ATCC). Authentication includes an assay to detect species specific variants of the cytochrome C oxidase I gene (COI analysis) to rule out inter-species contamination and short tandem repeat (STR) profiling to distinguish between individual human cell lines and rule out intra-species contamination.
Mycoplasma contamination	Routinely tested: negative
Commonly misidentified lines<br>(See ICLAC register)	N/A

Animals and other research organisms

Policy information about studies involving animals; ARRIVE guidelines recommended for reporting animal research, and Sex and Gender in Research

Laboratory animals	6- to 12-week old male and female NOD Scid Gamma (NSG) mice of similar weights
Wild animals	This study did not involve wild animals.
Reporting on sex	Findings apply to both sexes
Field-collected samples	This study did not involve samples collected from the fields.
Ethics oversight	All experimental procedures described in this study were conducted in accordance with relevant institutional and international

Ethics oversight	ethical guidelines and regulations.<br>For the CDX experiments, protocols were approved by the Animal Experimentation Ethics Committee (CEEA) of the University of Granada and conducted in accordance with the guidelines of the University's Bioethics Committee and the Guide for the Care and Use of Laboratory Animals. All procedures were performed under isoflurane inhalation anesthesia, and every effort was made to minimize suffering. Mice were kept in cages under a 12-hour light/dark cycle, 20-24 °C, 45-65% relative humidity with food and water available ad libitum. No more than five mice were housed in each cage.

Note that full information on the approval of the study protocol must also be provided in the manuscript.

Flow Cytometry

Plots

Confirm that:

☒ The axis labels state the marker and fluorochrome used (e.g. CD4-FITC).

☒ The axis scales are clearly visible. Include numbers along axes only for bottom left plot of group (a 'group' is an analysis of identical markers).

☒ All plots are contour plots with outliers or pseudocolor plots.

☒ A numerical value for number of cells or percentage (with statistics) is provided.

Methodology

Sample preparation	Cells were harvested from culture, washed and resuspended in cold 1X PBS.
Instrument	BD FACS Canto II
Software	FlowJo v10.7
Cell population abundance	Positively-selected cells (Atto550 or GFP) ranged accounted for >80% of total events
Gating strategy	First, bulk population of cells was selected by opposing FSC-A vs SSC-A. Then, SSC-A vs SSC-H was used to exclude doublets. Finally, GFP (FITC) and Atto550 (PE-A) positive populations were gated by comparing with negative controls.

☒ Tick this box to confirm that a figure exemplifying the gating strategy is provided in the Supplementary Information.
